# Polysaccharides as Stabilizers for Polymeric Microcarriers Fabrication

**DOI:** 10.3390/polym13183045

**Published:** 2021-09-09

**Authors:** Tatiana S. Demina, Liubov A. Kilyashova, Tatiana N. Popyrina, Eugenia A. Svidchenko, Sankarprasad Bhuniya, Tatiana A. Akopova, Christian Grandfils

**Affiliations:** 1 Enikolopov Institute of Synthetic Polymeric Materials, Russian Academy of Sciences, 70 Profsouznaya Str., 117393 Moscow, Russia; popyrina@ispm.ru (T.N.P.); evgensv@yandex.ru (E.A.S.); akopova@ispm.ru (T.A.A.); 2 Institute for Regenerative Medicine, Sechenov First Moscow State Medical University (Sechenov University), 8-2 Trubetskaya Str., 119991 Moscow, Russia; 3 Moscow Aviation Institute, National Research University, Orshanskaya Str. 3, 121552 Moscow, Russia; lyuba-97@mail.ru; 4 Centre Interdisciplinary Sciences of the JIS Institute of Advanced Studies and Research (JISIASR), JIS University, Arch Waterfront, GP Block, Sector V Bidhannagar, Kolkata 700091, West Bengal, India; spbhuniya@gmail.com; 5 Interfaculty Research Centre on Biomaterials (CEIB), Chemistry Institute, University of Liège, B6C, 11 Allée du 6 août, Sart-Tilman, B-4000 Liege, Belgium; c.grandfils@uliege.be

**Keywords:** microcarriers, polysaccharides, oil/water emulsions, interface, Pickering emulsions, stabilization

## Abstract

Biodegradable polymeric microparticles are widely used in drug delivery systems with prolonged-release profiles and/or cell microcarriers. Their fabrication via the oil/water emulsion solvent evaporation technique has normally required emulsifiers in the aqueous phase. The present work aims to evaluate the effectiveness of various polysaccharides, such as chitosan, hyaluronic acid, cellulose, arabinogalactan, guar and their derivatives, as an alternative to synthetic surfactants for polylactide microparticle stabilization during their fabrication. Targeted modification of the biopolymer’s chemical structure was also tested as a tool to enhance polysaccharides’ emulsifying ability. The transformation of biomacromolecules into a form of nanoparticle via bottom-up or top-down methods and their subsequent application for microparticle fabrication via the Pickering emulsion solvent evaporation technique was useful as a one-step approach towards the preparation of core/shell microparticles. The effect of polysaccharides’ chemical structure and the form of their application on the polylactide microparticles’ total yield, size distribution and morphology was evaluated. The application of polysaccharides has great potential in terms of the development of green chemistry and the biocompatibility of the formed microparticles, which is especially important in biomedicine application.

## 1. Introduction

The widespread application of biopolymers and green chemistry principles is a rational trend in modern sustainable materials science. The screening of polymers of natural origin for their potential to replace synthetic polymers used for emulsion formulations is an important part of this trend [1]. Indeed, a significant area of the food industry, pharmacy, and biomedicine rely on various types of emulsions. Stabilization of the interface is a key process in the fabrication of drug delivery systems and a range of materials tailored for biomedicine. For example, polymeric nano/microcarriers are widely used as drug delivery systems or multifunctional cell microcarriers [2,3,4,5,6,7]. One of the most popular methods of biodegradable particle fabrication is the oil/water emulsion solvent evaporation technique, which is based on the evaporation of solvents from the oil phase consisting of a core polymeric solution and transformation of the liquid-in-liquid emulsion into a suspension of solid particles within the aqueous phase [7,8,9,10]. The aqueous phase normally contains an emulsifier to prevent phase separation and particle aggregation during their fabrication. Widely used synthetic emulsifiers, such as polyvinyl alcohol (PVA), reside on the interface even after the particle’s solidification, and thus do not present benefits for their future application in view of their biodegradability and bioactivity [11]. Therefore, replacing a synthetic emulsifier with a naturally derived one could be beneficial in terms of control over the microparticle surface’s chemical structure and properties, which is a key feature of their application as cell microcarriers. Biodegradable microparticles are mainly fabricated from polyesters, such as polylactide (PLA), poly(lactide-co-glycolide) (PLGA), polycaprolactone (PCL), etc., which possesses many advantages from the point of view of processability, mechanical properties, and biodegradability, but could not provide enough biocompatibility for cell adhesion and spreading. To enhance microcarrier biocompatibility, a large number of post-treatment techniques of coating deposition were proposed [12]. The application of biopolymers as emulsifiers in the aqueous phase could be a more time- and effort-saving approach for enriching the microparticle surface by bioactive moieties in situ.

Several reported works showed the effectiveness of various biopolymers, i.e., polysaccharides and proteins, for emulsion stabilization [1,13,14,15]. Biopolymers also successfully used for the fabrication of Pickering emulsions, i.e., ones stabilized by the nanoparticles or nanogels. In contrast to traditional surfactants, nanoparticles could be even more effective for interface stabilization even at a low concentration [16,17,18]. Biopolymer-based nanoparticles could be fabricated using a variety of methods. The well-known “top-down” approach to polysaccharide nanoparticle fabrication is based on selective hydrolysis of amorphous regions within semi-crystalline polysaccharides, such as cellulose, chitin, etc., which led to the isolation of separate crystalline nanoparticles [19,20]. Polysaccharide nanocrystals are a popular form of polysaccharide nanoparticles that could be also used for emulsion interface stabilization [21,22]. Another approach, i.e., “bottom-up”, is based on chitosan macromolecule self-aggregation or complexation [23]. A few works were focused on the fabrication of polymeric particles via Pickering emulsions stabilization by polysaccharide-based nanoparticles [24,25,26]. A tough shell of nanoparticles led to high resistance to coalescence during emulsification, giving rise to core/shell microparticles, which are very promising for various applications [27]. Therefore, the Pickering emulsion approach could be particularly interesting for the fabrication of polymeric core/shell particles with tailored structure/properties.

This work aimed at the wide screening of polysaccharides and their derivatives in the form of macromolecular solutions and nanoparticles (nanogels) fabricated via bottom-up and top-down approaches as emulsifiers for the fabrication of polylactide microparticles via the oil/water solvent evaporation technique.

## 2. Materials and Methods

### 2.1. Material

Guar and hydroxypropyl guar (HPG), which mainly consist of high-molecular weight (50 kDa–8 MDa) polysaccharides composed of galactomannans (or their hydroxypropyl ethers); a mannose:galactose ratio of about 2:1; hydroxyethyl cellulose (HEC) of L250 Natrosol with a molecular weight (Mw) of 90 kDa and HEC of 250HHBR Natrosol with Mw of 1.3 MDa, were obtained from Ashland, Ashland, OH, USA. Arabinogalactan (AG) with an Mw of 24 kDa was purchased from VitaRost (Moscow, Russia). Iota-carrageenan (IC) and maltodextrin were food grades. Sodium hyaluronate (HA) with an Mw of 2.32 MDa was purchased from Bloomage Biotechnology (Jinan, China). Chitosan marked Chs-350/0.86 with an Mw of 350 kDa and a degree of deacetylation (DD) of 0.86 was purchased from “Sonat” (Moscow, Russia). Chitosan Chs-60/0.90 with an Mw of 60 kDa and a DD of 0.90 and chitosan Chs-80/0.87 (Mw of 80 kDa, DD of 0.87) were produced from chitin via mechanochemical alkaline deacetylation as described previously [28]. Derivatives of chitosan Chs-60/0.90 N-acetylated by 2,2-bis(hydroxymethyl)propionic acid were synthesized and characterized as reported earlier [29]. These derivatives were of a different degree of chitosan amino group substitution, i.e., 0.16, 0.18, 0.24 and 0.43, and were marked as ChsB-0.16, ChsB-0.18, ChsB-0.24 and ChsB-0.43, respectively. Polylactide (PLA, Natureworks 4043D (Minnetonka, MN, USA)) with an Mw of 100 kDa was used as a core material for polymeric microparticle preparation. All solvents were from Chimmed (Moscow, Russia).

Polysaccharide nanoparticles were fabricated using “bottom-up” and “top-down” approaches. The first one was realized by the controlled precipitation of chitosan Chs-60/0.90 and its N-acetylated derivatives. Briefly, chitosan or its derivative was dissolved either in 2%CH_3_COOH (non-modified chitosan Chs-60/0.90) or in distilled water (ChsB samples) to form 2 wt.% solution; then, 1M NaOH was added dropwise within these solutions under a constant agitation to achieve pH 6.5 (chitosan’s pKa). Then, these half-precipitated solutions were subjected to ultrasound treatment at a frequency of 23 kHz for 3 min. The obtained chitosan/derivative suspensions were marked as the respective polymer with the -NP suffix (for example, Chs-60/0.90-NP). To confirm precipitation of chitosan gel nanoparticles, UV/Vis-spectroscopy and dynamic laser scattering (DLS) of initial chitosan/derivatives solutions and their precipitated forms were realized. UV/Vis-spectroscopy was carried out in a quartz cell with an optical path length of 1 cm using a Shimadzu UV 2501 PC spectrophotometer. DLS was realized with using a Zetatrac particle size analyzer (Microtrac, Inc., Montgomeryville, PA, USA) with the software program V.10.5.3. A “top-down” approach to fabrication of polysaccharide nanoparticles was realized by means of the isolation of nanocrystals from cellulose or chitin samples to form cellulose nanocrystals (Cel-NC) from bleached flax stalk cellulose and chitin nanocrystals from a chitin sample purchased from “Kombio” (Vladivostok, Russia) (Ch-K-NC) or “Xiamen Fine Chemical” (Xiamen, China) (Ch-X-NC), as was reported earlier [25,30,31].

### 2.2. Microparticle Fabrication and Characterization

The ability of various polysaccharides to stabilize an oil/water interface was evaluated in the course of the fabrication of PLA microparticles via the oil/water emulsion solvent evaporation technique according to the methodology described previously [31,32]. Briefly, the oil phase consisting of a 5 wt.% PLA solution in a solvent mixture of CH_2_Cl_2_:acetone = 9:1 *v*/*v* was rapidly added to an aqueous phase; the oil/water phase ratio was 9/1 *v*/*v*. The aqueous phase consisted of a 1 wt.% solution of biopolymers in distilled water, except for collagen and non-modified chitosan samples, which were dissolved in 2%CH_3_COOH. Solutions of HA were preliminarily treated by ultrasound at 23 kHz for 3 min. The oil and aqueous phases were mixed using a 4-bladed propeller stirrer at 700 rpm. The systems were thermostated at 15 °C within the first 15 min after the addition of the oil phase, then the temperature was increased up to 30 °C to promote the evaporation of organic solvents from the oil phase. The fabricated microparticles were washed with distilled water, sieved with apertures ranging from 100 to 400 µm, and freeze-dried.

Microparticle morphology was evaluated using scanning electron microscopy (SEM) using PhenomProX (ThermoFisher, Waltham, MA, USA)

## 3. Results

### 3.1. Non-Modified Biomacromolecules as Emulsifiers for PLA Microparticle Fabrication

**Figure 1:**
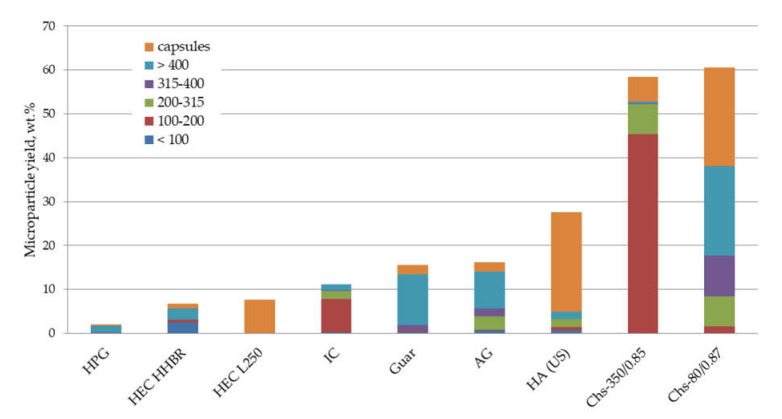
Total yield and size distribution of PLA microparticles stabilized with various polysaccharides in the course of their fabrication via the oil/water solvent evaporation technique. The legend shows a size range (μm) of microparticle fractions.

The data presented in Figure 1 show the ability of various polysaccharides to stabilize the oil/water interface in the course of PLA microparticle fabrication, which refers to the microparticle total yield and their size. Maltodextrin failed to stabilize the interface at all, and thus the PLA dissolved in the oil phase aggregated. Other nonionic polysaccharides possessed a weak ability as emulsifiers, regardless of their molecular weight. A better ability to stabilize PLA microparticles was observed in the case of polysaccharides containing ionogenic groups, i.e., hyaluronic acid and chitosan. Two chitosan samples with different Mw both stabilized up to 58–60 wt.% of the PLA dissolved in the oil phase. The Mw of chitosan affected the size distribution of the formed microparticles, without any significant effect on total yield.

### 3.2. Effect of Chitosan Chemical Structure on Its Emulsification Ability

**Figure 2:**
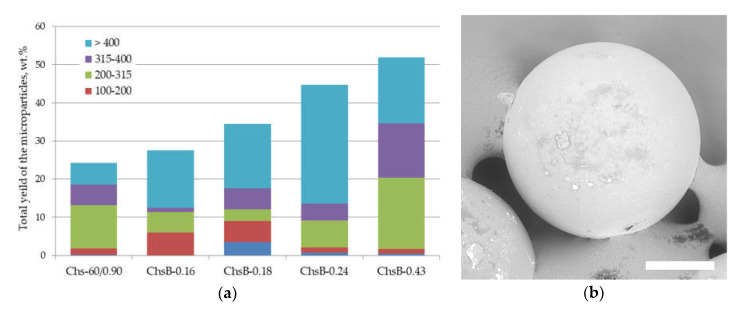
(**a**) Total yield and size distribution of PLA microparticles stabilized with 2 wt.% solutions of chitosan Chs-60/0.90 and its ChsB derivatives; (**b**) SEM micrograph of the obtained PLA microparticles. Scale bar is 100 μm.

The effect of the modification of chitosan’s chemical structure on its ability to stabilize the interface was evaluated using a range of N-acetylated 2,2-bis(hydroxymethyl)propionic acid derivatives of chitosan Chs-60/0.90. We also studied the effect of polymer concentration dissolved in the aqueous phase, which showed a logical increase in the total yield of the microparticle as a function of the polymer concentration increase: a 2 wt.% concentration was found to be sufficient (Supplementary Figure S1). Figure 2 shows the microparticle total yield as a function of the degree of chitosan amino group substitution. The total yield was increased from 24 wt.% (non-modified chitosan Chs-60/0.90) to 52 wt.% (ChB-0.43, the chitosan derivative with a degree of substitution of 0.43). As can be seen in Figure 2b, the fabricated microparticles had a spherical shape and a homogenous surface morphology.

### 3.3. Core/Shell Microparticles Stabilized by Biopolymer Nanoparticles

#### 3.3.1. Microparticles Stabilized by Chitosan Nanoparticles Obtained by Polysaccharide Precipitation

**Figure 3:**
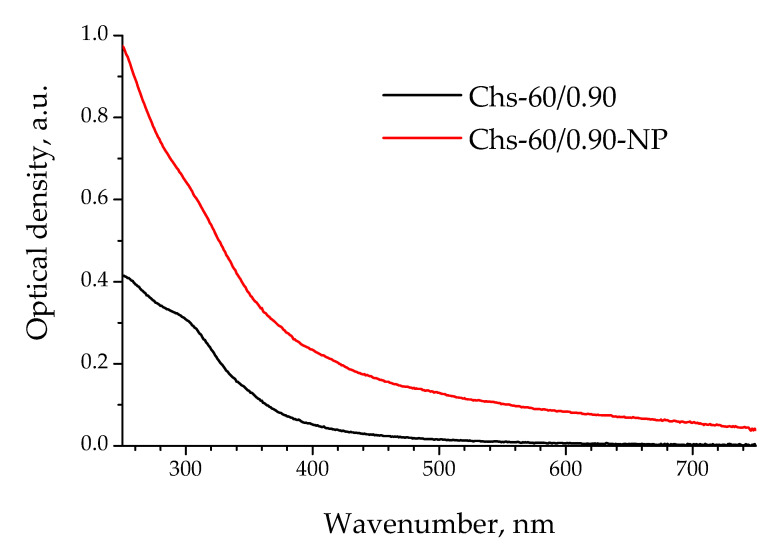
Electron absorption spectra of 0.5 wt.% chitosan Chs-60/0.90 solution in 2%CH_3_COOH in and nanoparticles Chs-60/0.90-NP fabricated by chitosan precipitation.

Chitosan-based gel nanoparticles were prepared by their controlled precipitation from aqueous acetic acid solution by alkali. A comparison of UV/Vis-spectra of the chitosan Chs-60/0.90 solution and the nanoparticles obtained by its precipitation via increasing pH to 6.5 is shown in Figure 3. The optical density of the precipitated polymer was higher than that of the initial chitosan solution in the whole studied range from 200 to 800 nm, which indicated the formation of polymer associates.

**Figure 4:**
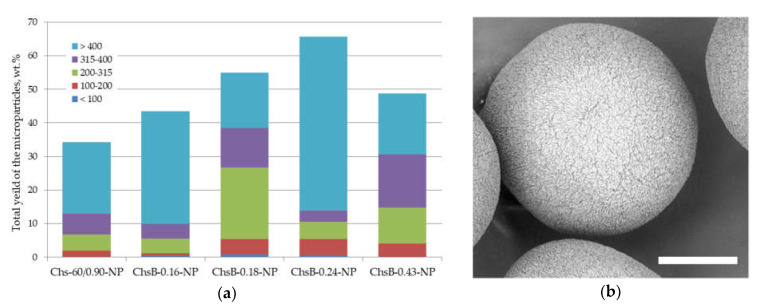
(**a**) Total yield and size distribution of PLA microparticles stabilized with nanoparticles obtained by the precipitation of 2% solutions of chitosan Chs-60/0.90-NP and its ChsB derivatives; (**b**) SEM micrograph of PLA microparticles stabilized with precipitated chitosan nanoparticles. The scale bar is 100 μm.

The histogram of the total yield and size distribution of fabricated PLA microparticles using the precipitated chitosan-based nanogels as emulsifiers in the aqueous phase is shown in Figure 4a. The total yield was significantly higher than that in the case of the application of respective non-modified chitosan and its ChsB derivatives in the form of a solution. The yield was increased when the degree of amino group substitution was increased up to DS of 0.24/0.43. The application of nanoparticles obtained by precipitation of chitosan derivatives with higher DS led to an increase in the mean size of the fabricated PLA microparticles (see ChsB-0.24-NP) and a decrease in their total yield (ChsB-0.43-NP). Figure 4b presents a micrograph of PLA microparticles stabilized with nanoparticles obtained by the precipitation of chitosan Chs-60/0.90.

#### 3.3.2. Application of Polysaccharide Nanocrystals Fabricated via Top-Down Approach

**Figure 5:**
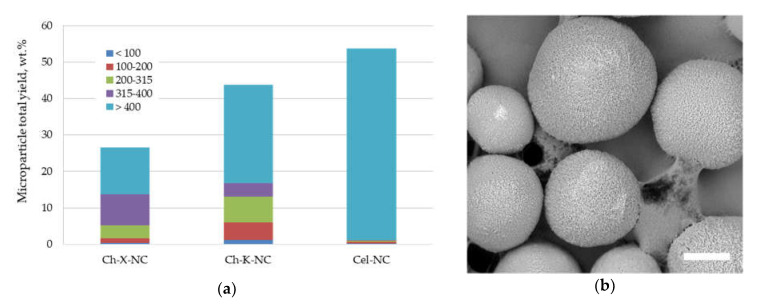
(**a**) Total yield and size distribution of PLA microparticles stabilized with 0.1 wt.% water dispersions of polysaccharide nanocrystals of various origins: the legend shows a size range (μm) of microparticle fractions; (**b**) SEM micrograph of PLA microparticles stabilized with 0.1 wt.% of Ch-K-NC water dispersion. The scale bar is 100 μm.

The effect of polysaccharide nanocrystals’ origin on the total yield and size distribution of the fabricated PLA microparticles using water dispersions of these nanocrystals as the aqueous phase is shown in Figure 5a. The total yield of PLA microparticles stabilized with cellulose nanocrystals (Cel-NC) was higher than that of those prepared using chitin nanocrystals (Ch-X-NC and Ch-K-NC). However, the mean size of Cel-NC-stabilized microparticles was significantly larger than that of the PLA particles stabilized with chitin nanocrystals.

## 4. Discussion

Within the scope of our work, we focused on the applicability of various polysaccharides for the stabilization of polylactide particles in the course of their fabrication via the oil/water emulsion solvent evaporation technique. As could be seen from the screening of a range of biopolymers as emulsifiers in the aqueous phase, some of the polysaccharides allowed us to fabricate PLA microparticles with high total yields and a small mean size (Figure 1). The ability to stabilize the microparticles was correlated with a biopolymer emulsification ability reported in the literature [1]. Among polysaccharides, hyaluronic acid and chitosan were found the most promising, in spite of these belonging to the non-absorbing types of emulsifiers. The best stabilization ability was found for chitosan, which has been widely studied for a range of biomedical applications [33,34,35]. However, the practical application of chitosan for regenerative medicine is limited to wound-dressing due to safety issues, including the potential immune response. It is interesting to note that the emulsifying ability of chitosan samples depends on the Mw and DD. Indeed, these chitosan characteristics have predominant effects on the polysaccharides’ emulsifying properties, but in spite of extensive studies, the relationship between Mw/DD and the ability of chitosan to stabilize the interface is not totally clear [13,36]. All chitosan samples used in our work had relatively high DD, which is a range of DD (approx. 0.80–0.89) shown to demonstrate good emulsifying activity when sunflower oil is used as an oil phase [36,37]. As can be seen in Figure 1, chitosan is also a good emulsifier in comparison with other polysaccharides in the case when the PLA solution is used as an oil phase. The significant difference in chitosan samples used in our work was their Mw. According to the literature data, the emulsifying activity of chitosan was higher when the Mw increased [36]. Chs-60/0.90, having the lowest Mw compared to Chs-350/0.85 and Chs-80/0.87, showed the lowest total yield of stabilized microparticles, in spite of increasing the concentration of the polymer in the aqueous phase up to 2 wt.%. We assumed that chitosan with a relatively low Mw could be more effective as an emulsifier when its chemical structure is modified.

Figure 2 shows a histogram of the total yield and size distribution of PLA microparticles stabilized by Chs-60/0.90 or its N-acetylated derivatives. The increase in total yield was increased two-fold when almost half of the chitosan amino groups were acylated by 2,2-bis(hydroxymethyl)propionic acid. Substitution of amino groups could be beneficial in terms of chitosan’s emulsifying ability from different points of view. At first, the high content of positively charged amino groups provides a steric stabilization mechanism to chitosan. On the other hand, hydrophobic fragments, including N-acetyl glucosamine units within initial chitosan, promote the polysaccharide emulsifying activity as well [36]. Therefore, partial substitution of NH_2_ groups could be used to regulate the hydrophilic/lipophilic balance of the macromolecule. Previously, we also successfully used grafting of hydrophobic oligolactide fragments onto chitosan to enhance its ability to stabilize an oil/water interface during the fabrication of PLA microparticles [32,38]. It is interesting to note that the combination of chitosan with a hydrophilic polysaccharide with a relatively low emulsifying activity, i.e., HEC, within one macromolecule was also shown to be an effective tool to fabricate a good bioemulsifier [39]. It seems that an enhanced stabilization ability of the copolymers consisting of polymers with different solubility could be caused by the superposition of various mechanisms of interface stabilization. One of these mechanisms is the formation of macromolecular associates within copolymer solutions and, therefore, the stabilization of an interface by macromolecular nanogel particles rather than by dissolved macromolecules.

A range of particles were tested to explore the effectiveness of polysaccharide nanoparticles as emulsifiers in the aqueous phase. Figure 4 shows the total yield and size distribution of PLA microparticles stabilized with polysaccharide nanoparticles obtained via a bottom-up approach, i.e., through the precipitation of chitosan Chs-60/0.90 or its N-acetylated derivatives. As polyelectrolyte chitosan dissolves when its amino groups are protonated, an increase in pH up to its pKα led to macromolecule precipitation along with the formation of nanogels [23]. The UV/Vis spectra of the chitosan solution and respective nanoparticles show a higher optical density within the wide wavelength range at increased pH, confirming the formation of nanoparticles (Figure 3). Non-modified chitosan in the form of nanoparticles allowed us to stabilize 34 wt.% of PLA dissolved in the oil phase, while the total yield of PLA particles stabilized with this chitosan in the form of a solution was only 24 wt.%. This effect is in good agreement with the well-known superior stability of Pickering emulsions in comparison with classical ones stabilized with molecular solutions [16]. This tendency was also observed in the case of the investigated ChsB derivatives. However, the effect of the macromolecular chemical structure on the total yield and size distribution of nanoparticle-stabilized PLA microparticles was more complex than that in the case of solution form. A significant decrease in the emulsifying ability of nanoparticles fabricated by the precipitation of highly acetylated ChsB-0.43 derivatives could be linked to their bigger size due to the introduction of a large number of volume substituents.

Polysaccharide nanoparticles fabricated via the top-down approach, i.e., polysaccharide nanocrystals, also have a number of characteristics which determine their ability to stabilize the oil/water interface. The morphology and chemical structure of the nanoparticle surface are key factors, and both depend on the features of the initial polysaccharide and the conditions of amorphous regions’ hydrolysis, as well as the crystalline domain. Nanocrystals’ chemical structures are responsible for their interaction at the interface and their tendency to aggregate within the aqueous phase. Figure 5a shows the total yield of PLA microparticles fabricated using nanocrystal water dispersions at a low concentration (0.1 wt.%). Although the cellulose nanocrystals (Cel-NC) seemed to be a rather promising stabilizer in terms of total yield, the fabricated PLA particles mostly had a size above 400 μm, which shows their limited ability to serve as an emulsifier. Moreover, the application of Cel-NC at higher concentrations (0.5–1 wt.%) led to nanocrystal aggregation within the water dispersion, which drastically decreased their ability to the stabilize oil/water interface, as was previously shown [31]. On the other hand, both types of chitin nanocrystals were able to form microparticles with a lower mean size, indicating the relatively good ability to stabilize the interface (Figure 2). This ability was also increased with an increase in nanocrystals concentration in the aqueous phase [25].

As can be seen in Figure 4b and Figure 5b, the surface morphology of the PLA microparticles fabricated via Pickering emulsion was heterogeneous, both in the case of nanocrystal-stabilized microparticles and the application of nanoparticles formed using the bottom-up approach, i.e., precipitated chitosan macromolecules. Apparently, this type of surface morphology is a specific feature of microparticles fabricated via the oil/water Pickering emulsion solvent evaporation technique, and it could be used to fabricate core/shell microparticles.

## Data Availability

Data is contained within the article.

## Supplementary Materials

The following are available online at https://www.mdpi.com/article/10.3390/polym13183045/s1, Figure S1: Total yield and size distribution of PLA microparticles stabilized with N-acetylated 2,2-bis(hydroxymethyl)propionic acid derivative of chitosan (sample ChsB-0.43) dissolved in 2%CH_3_COOH at various concentrations.
