# Supplementary material for: Polysaccharides as Stabilizers for Polymeric Microcarriers Fabrication
Source: Polymers (Basel). 2021 Sep 9;13(18):3045. doi: 10.3390/polym13183045 (PMC8467260; doi:10.3390/polym13183045)
Supplement: Supplementary file 1 [file polymers-13-03045-s001.zip › polymers-1362223-supplementary.pdf]

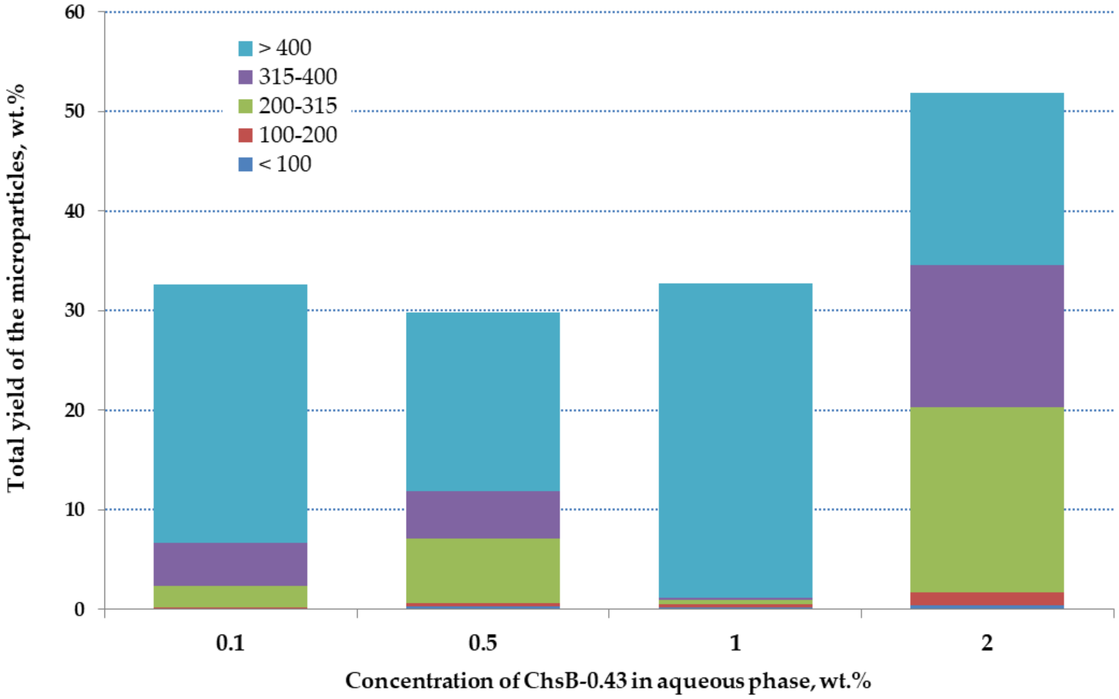

Figure S1. Total yield and size distribution of PLA microparticles stabilized with N-acetylated 2,2-bis(hydroxymethyl)propionic acid derivative of chitosan (sample ChsB-0.43) dissolved in 2%CH_3COOH at various concentrations.
